# Comparative Efficacy of Different Amoxicillin Dosing Regimens in Preventing Early Implant Failure—A Systematic Review with Network Meta-Analysis

**DOI:** 10.3390/antibiotics12030512

**Published:** 2023-03-03

**Authors:** Lee Wen Tan, Yan Er Ng, Koay Chun Giok, Sajesh K. Veettil, Rohit Kunnath Menon

**Affiliations:** 1 School of Dentistry, International Medical University, Kuala Lumpur 57000, Malaysia; 2 Research Associate, Department of Pharmacotherapy, College of Pharmacy, University of Utah, Salt Lake City, UT 81142, USA; 3 Prosthodontics, School of Dentistry, Ajman University, Ajman 346, United Arab Emirates; 4 Center of Medical and Bio-Allied Health Sciences Research, Ajman University, Ajman 346, United Arab Emirates

**Keywords:** implant failure, early implant failure, review, systematic review, meta-analysis

## Abstract

This systematic review and network meta-analysis aimed to assess the comparative efficacy and safety of antibiotics to prevent early implant failure in patients undergoing dental implant surgery. Methods: The review was registered in PROSPERO [CRD42022319385]. A search was conducted for trials published in Medline, Cochrane, PubMed, and Scopus. A network meta-analysis was performed on the data from randomized controlled trials. Agents were ranked according to their effectiveness based on outcomes (implant failure, prosthetic failure, postsurgical complications, and adverse effects) using the surface under the cumulative ranking [SUCRA]. Results: A total of 15 articles were included in the quantitative analysis. When compared to the placebo, 2 g of amoxicillin given 1 h preoperatively (RR = 0.42 (95%CI: 0.27, 0.67)), 2 g of amoxicillin given 1 h preoperatively with postoperative 500 mg thrice for 5 days (RR = 0.36 (95%CI: 0.15, 0.87)), and post-operative amoxicillin with clavulanic acid 625 mg 3 times daily for 5 days (RR = 0.38 (95%CI: 0.16, 0.90)) were effective in reducing early implant failures. In addition, 2 g of amoxicillin given 1 h preoperatively (RR = 0.42 (95%CI: 0.25, 0.73)) was the only protocol that was significant in the pairwise meta-analysis results. However, sensitivity analysis, which excluded trials with a high risk of bias, showed that none of the protocols were statistically significant in reducing early implant failure. Conclusions: A single 2 g dose of preoperative amoxicillin significantly reduces early implant failure in healthy individuals. More high-quality trials are required to establish this recommendation, as the quality of this evidence is weak.

## 1. Introduction

Dental implant failure may occur before prosthetic loading (early) due to inadequate osseointegration or after loading (late) [1,2]. Early implant failures may be due to impaired healing due to the micromovement of components, impaired bone quality, contamination of the implant surface, mechanical factors, or patient-related factors like smoking [3,4,5,6,7,8,9]. Osseointegration of dental implants requires meticulous oral hygiene practices with chlorhexidine rinses as an adjunctive measure to promote healing and prevent infection [10,11]. Systemic antibiotics are routinely prescribed in implant surgery with the goal of preventing infection and, consequently, early implant failures [12]. Amoxicillin is the preferred antibiotic, and a single preoperative dose of amoxicillin has been deemed as effective in reducing implant failure rates [13,14]. However, a standardized protocol for the administration of amoxicillin has not yet been established, prompting researchers to suggest further research to establish one [15,16]. The establishment of a standardized protocol is essential to avoid the overuse of antibiotics and their associated detrimental effects, including antibiotic resistance [17]. The long-term impact of amoxicillin on the oral microbiome has been recognized previously by next-generation sequencing research [18,19]. Even though recent systematic reviews suggest the use of preoperative antibiotics for the prevention of early implant failures, there is no conclusive evidence on the type, dose, timing, and duration of antibiotic treatment [15,16,20]. Moreover, current evidence is inconclusive on the harm-benefit aspect of antibiotic treatment for dental implant surgery [21,22]. Unlike conventional pairwise meta-analysis, network meta-analysis (NMA) enables investigators to combine direct and indirect evidence to establish comparative efficacy and acceptability across a network of randomized controlled trials (RCTs) of all interventions until now. Choosing the most efficacious antibiotic regimen for preventing early implant failure is an important consideration in dentistry. Hence, this study aims to perform a NMA to assess the comparative effectiveness of interventions used to prevent early implant failure. This study aims to recommend an appropriate antibiotic dosage regime that is significant in preventing early implant failure while justifying the impact on the oral microbiome and the risk of antibiotic resistance [23]. 

## 2. Materials and Methods

A systematic review of randomized controlled trials comparing interventions for the prevention of early implant failure in patients undergoing dental implant surgery was undertaken according to the general principles outlined in *The Cochrane Handbook for Systematic Reviews of Interventions* and reported according to the Preferred Reporting Items for Systematic Reviews and Meta-Analyses (PRISMA) extension statement for NMA [24,25]. The proposal for this systematic review was registered in the PROSPERO registry [CRD42022319385]. We identified relevant studies through a systematic search of Medline, Cochrane, PubMed, and Scopus. In addition, published systematic reviews were searched for relevant randomized controlled trials. 

### 2.1. Search Strategy and Study Selection

A search of human studies in these databases from inception through 6 October, 2022, was performed by using subject headings and free text terms. Three sets of search terms were combined: terms for implant failure, terms for the relevant intervention (systemic antibiotics), and a search filter to identify randomized controlled trials. We developed the search strategy for MEDLINE and modified it for other databases. In vitro studies, studies that were not in English, animal studies, summary reviews, systematic reviews, and articles with unclear data were not included. Two of the authors performed the search within the mentioned databases.

### 2.2. Outcomes of Interest

Studies included were randomized controlled trials (RCTs) that met the following inclusion criteria: participants will be anyone undergoing dental implant surgery; interventions are systemic antibiotics; comparisons are placebo, no treatment, or any other active intervention; and the primary outcome is early implant failure. Secondary outcomes are surgical site infection and adverse effects. The criteria for early implant failure deployed by the studies selected were instability of implant [26,27,28,29,30], removal of implant due to infection [31,32,33], removal of implant for any reason from implant placement to abutment connection or prosthetic treatment [34], mobility of implant or removal due to pain or infection [35,36], presence of mobility or infection or instability of implant [37,38], and the presence of mobility of implant [39,40]. There was a high degree of heterogeneity across all the studies in the case definition of early implant failure.

### 2.3. Data Extraction and Quality Assessment

Two authors independently screened titles and abstracts for eligible studies, followed by full-text reading. Ineligible studies were excluded from full-text review, and the reasons for exclusion were documented. Data was extracted independently and in duplicate by the three reviewers and entered into a data extraction form created following *The Cochrane Handbook of Systematic Reviews of Interventions* guidelines [24]. If multiple publications of the same trial were retrieved, only the most recent relevant data will be included from these publications. The data from RCTs were separated into the following sections: study characteristics, population characteristics, intervention characteristics, and outcome definitions and measures. For all outcomes, the initial number of participants randomized to each trial arm was used, and analyses were performed irrespective of how the authors of the original trials had analyzed the data (intention-to-treat principle). Two reviewers independently assessed the risk of bias (RoB) within each study by using the revised Cochrane risk of bias tool (RoB 2.0) [41]. Reviewers resolved disagreements through discussion. 

### 2.4. Data Synthesis and Statistical Analysis

Data was analyzed according to the intention to treat the principle as a primary analysis. Risk ratios and 95% confidence intervals was used as summary statistics. For direct comparisons, a standard pairwise meta-analysis was performed by using a random effects (DerSimonian and Laird) model [24]. If a direct comparison was based on two or more studies, heterogeneity between trials was assessed using I^2^ statistics; an I2 estimate ≥50% is interpreted as evidence of substantial levels of heterogeneity [24]. A random-effects network meta-analysis (frequentist approach) using either a consistency or an inconsistency model was applied to synthesize the available evidence by combining direct and indirect evidence from different studies [42,43]. The network inconsistency assumption, which refers to a disagreement between the direct and indirect estimates, was evaluated using a global inconsistency test by fitting design-by-treatment into the inconsistency model [44,45]. The probability of each treatment was estimated as being the best to construct rankograms (a relative ranking of interventions) and their surface area under the cumulative ranking (SUCRA) [46]. Publication bias was examined with a comparison-adjusted funnel plot [47]. For statistical analysis and graph generation, Stata version 15.0 (StataCorp, College Station, TX, USA) was utilized [46]. To assess the robustness of our primary efficacy outcomes, we performed multiple pre-specified sensitivity analyses by restricting studies with low risk of bias.

## 3. Results

**Figure 1:**
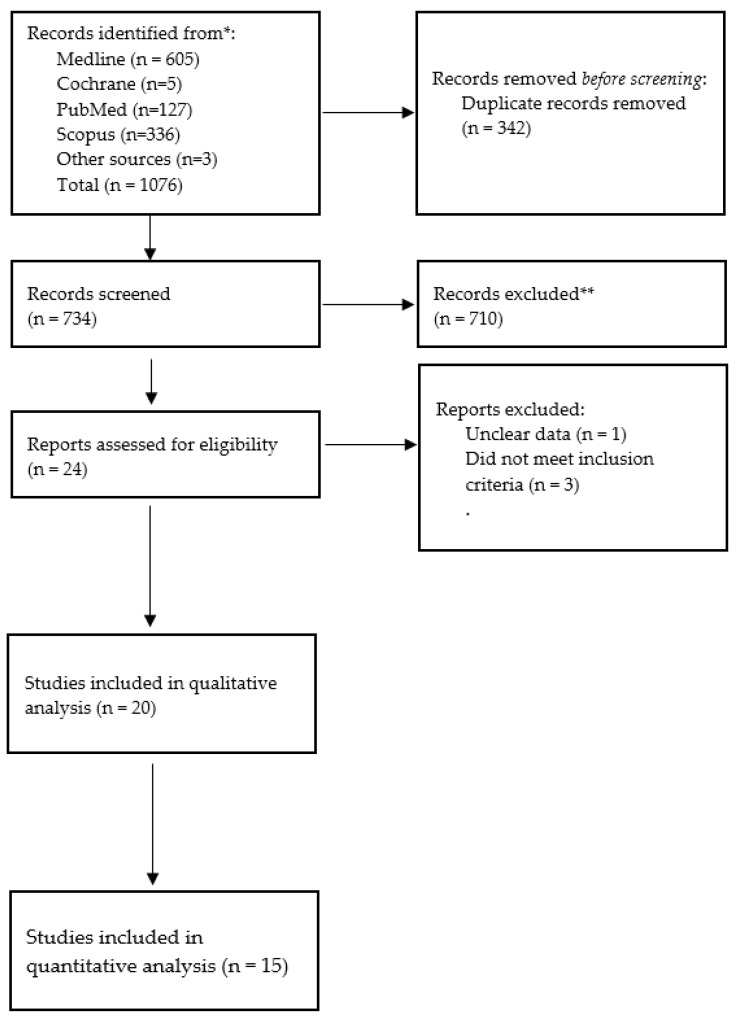
PRISMA flow diagram summarizing the study selection process for eligible trials. * Databases from which records were identified. ** Explanation of records excluded: two summary reviews; one with unclear data; and one that did not report the outcomes.

A total of 1076 articles were initially identified, and 342 duplicates were removed. A total of 710 articles were excluded by screening the titles and abstracts. A total of 24 articles were assessed by full-text reading, and finally 15 articles were included in the quantitative synthesis (Table 1). The PRISMA flow diagram is shown in Figure 1. 

The different dosing regimens of antibiotics varied from 1 h preoperatively to postoperatively for 7 days, and the time of evaluations following treatment ranged from 2 days to the 8th week for adverse effects and postsurgical complications, and from 10 days to 6 months for implant failure. A table that summarizes all of the abbreviations used in this current study can be accessed in Supplementary Table S1.

The quality assessment of each study using the RoB assessment tool is provided in Supplementary Table S2, and the reasons for the exclusion of four articles from the full text review are provided in Supplementary Table S3. Nine trials were evaluated to be at high RoB [26,27,31,34,35,36,37,39,40]; two studies were graded with some concerns [32,33]; and the remaining four studies had low bias [28,29,30,38].

### 3.1. Implant Failure (per Patient Data)

**Figure 2:**
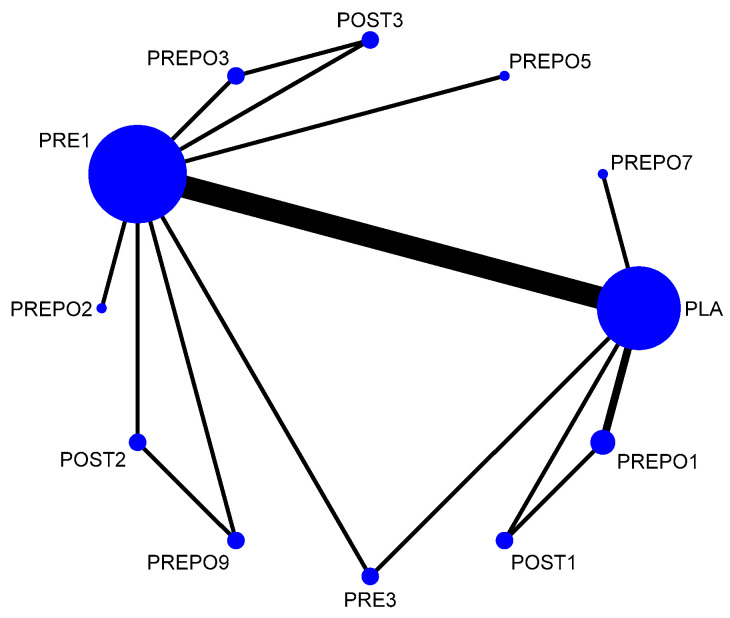
Network plot: implant failure (per patient). Abbreviations: PLA = placebo/no antibiotics; PRE1 = 2 g amox 1 h pre-op; PRE3 = 3 g amox 1 h pre-op; POST1 = 2 g amox immediately post-op; POST2 = post-op amox with clavulanic acid 625 mg 3 times daily for 5 days; POST3 = 500 mg q 8 h for 5 days post-op/postoperative amox 1 g twice a day for 1 week after surgery; PREPO1 = 2 g amox pre-op and 500 mg amox post-op for 3 days; PREPO2 = 2 g amox 1 h pre-op followed by 500 mg three times for 7 days; PREPO3 = 2 g amox 1 h pre-op, 500 mg q 8 h for 5 days; PREPO5 = 2 g amox pre-op and 1 g amox postoperative, and 1 g twice a day for 2 days later; PREPO7 = 1 g amox 1 h pre-op and 500 mg 4x daily for 2 days post-op; PREPO9 = 2 g amox 1 h pre-op, followed by postoperative 500 mg thrice for 5 days.

A total of 12 randomized control trials comparing 11 interventions (Figure 2) were included in the NMA, which is expressed as the risk ratio (RR) of implant failure per patient when treatment was given as compared to a placebo. When compared to the placebo, protocols POST2 (RR = 0.38(95%CI: 0.16, 0.90)), PRE1 (RR = 0.42(95%CI: 0.27, 0.67)), and PREPO9 (RR = 0.36(95%CI: 0.15, 0.87)) were statistically proven to be significant in preventing implant failure at the patient level. The protocol PREPO9 ranked first (SUCRA 66.3), followed by POST2 (SUCRA 64.0), and lastly PRE1 (SUCRA 59.6) in preventing implant failure.

**Figure 3:**
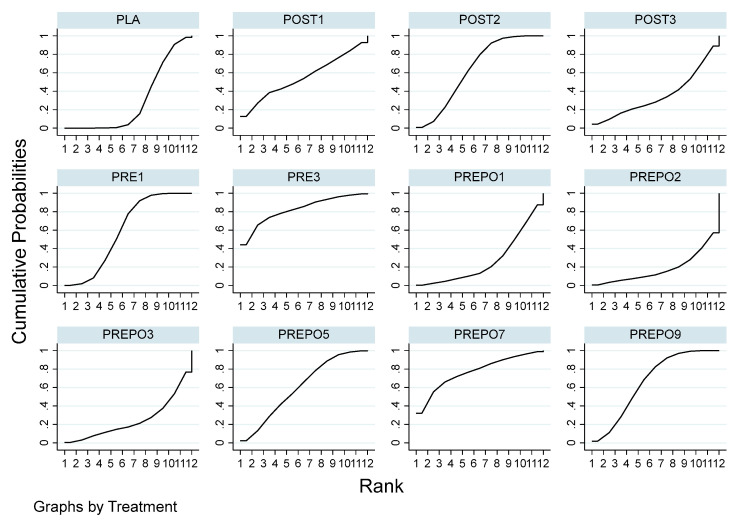
SUCRA ranking curve in implant failure (per patient). Abbreviations: PRE1 = 2 g amox 1 h pre-op; PRE3 = 3 g amox 1 h pre-op; POST1 = 2 g amox immediately post-op; POST2 = post-op amox with clavulanic acid 625 mg 3 times daily for 5 days; POST3 = 500 mg q 8 h for 5 days post-op/postoperative amox 1 g twice a day for 1 week after surgery; PREPO1 = 2 g amox pre-op and 500 mg amox post-op for 3 days; PREPO2 = 2 g amox 1 h pre-op followed by 500 mg three times for 7 days; PREPO3 = 2 g amox 1 h pre-op, 500 mg q 8 h for 5 days; PREPO5 = 2 g amox pre-op and 1 g amox postoperative, and 1 g twice a day for 2 days later; PREPO7 = 1 g amox 1 h pre-op and 500 mg 4x daily for 2 days post-op; PREPO9 = 2 g amox 1 h pre-op, followed by postoperative 500 mg thrice for 5 days.

Table 2 summarizes the relative risk (RR) and the ranking of the treatment options. Figure 3 shows the SUCRA ranking curves for each protocol.

**Figure 4:**
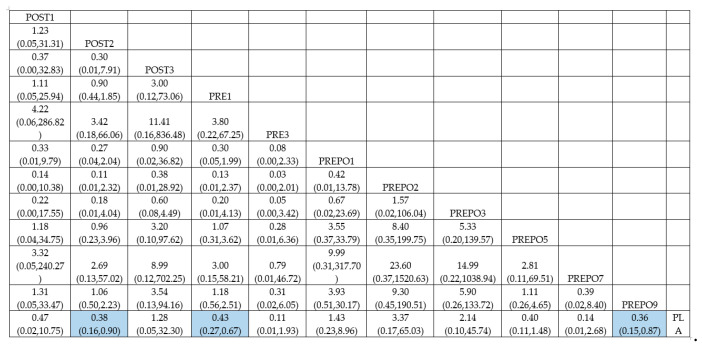
League table for implant failure (per patient).

The direct and network estimates for the efficacy of these agents are shown in Figure 4.

Pairwise meta-analysis was conducted for studies with direct comparisons, as shown in Supplementary Figure S1. It was shown that the intervention PRE1 (RR = 0.42(95%CI: 0.25, 0.73)) was statistically significant in preventing implant failure.

A sensitivity analysis was performed due to the high number of studies susceptible to a high risk of bias, as shown in Supplementary Figure S2. The analysis demonstrated no statistically significant protocols, as seen in Supplementary Table S3.

### 3.2. Implant Failure (per Implant)

**Figure 5:**
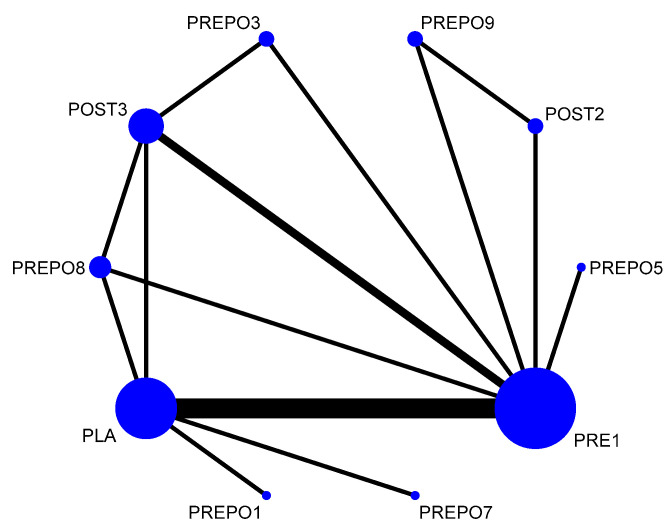
Network plot: implant failure (per implant). Abbreviations: PLA = placebo/no antibiotics; PRE1 = 2 g amox 1 h pre-op; POST2 = post-op amox with clavulanic acid 625 mg 3 times daily for 5 days; POST3 = 500 mg q 8 h for 5 days post-op/postoperative amox 1 g twice a day for 1 week after surgery; PREPO1 = 2 g amox pre-op and 500 mg amox post-op for 3 days; PREPO3 = 2 g amox 1 h pre-op, 500 mg q 8 h for 5 days; PREPO5 = 2 g amox pre-op and 1 g amox postoperative, and 1 g twice a day for 2 days later; PREPO7 = 1 g amox 1 h pre-op and 500 mg 4x daily for 2 days post-op; PREPO8 = 2 g amox 1 h pre-op, followed by postoperative 1 g amox twice a day for 7 days; PREPO9 = 2 g amox 1 h pre-op, followed by postoperative 500 mg thrice for 5 days.

Eleven randomized control trials investigating nine interventions were analyzed to assess the efficacy of a preoperative antibiotic in preventing implant failure per implant. The network plot derived is shown in Figure 5. The network estimates that only protocol PRE1 (RR = 0.49 (95%CI: 0.26, 0.92)) is statistically significant in preventing implant failure. 

**Figure 6:**
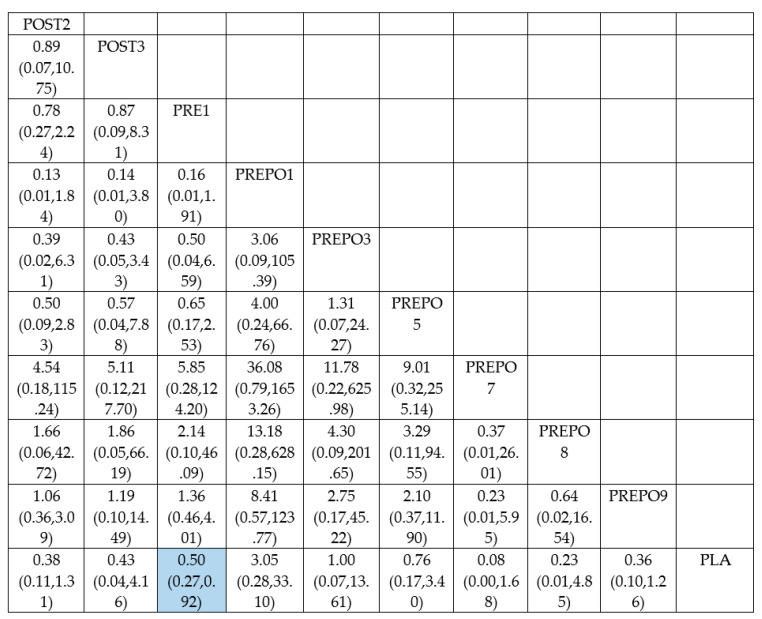
League table for implant failure (per implant).

Table 3 summarizes the RR and the SUCRA ranking of the treatment options, while statistically significant differences are listed in the league table (Figure 6).

**Figure 7:**
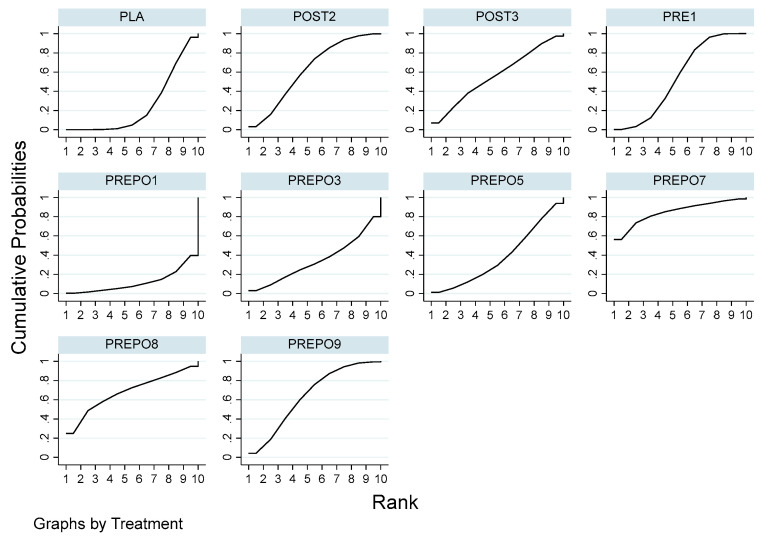
SUCRA ranking curve for implant failure (per implant).

The ranking of the efficacy of the interventions was based on SUCRA, and protocol PREPO7 ranked the highest. Only protocol PRE1 was statistically significant. The SUCRA ranking curves for each protocol are presented in Figure 7. These direct and network estimates of implant failure (implant) are presented in Figure 6.

Pairwise meta-analysis revealed no significant results, as shown in Supplementary Figure S3.

### 3.3. Prevention of Prosthetic Failure

Three randomized control trials investigating two protocols were analyzed to assess the efficacy of the preoperative antibiotic in preventing prosthetic failure. The network plot derived is shown in Supplementary Figure S4. The network estimates show that, compared to the placebo, none of the protocols were estimated to be statistically significant. Supplementary Table S4 and Supplementary Figure S5 summarize the RR and the SUCRA ranking of the treatment options. The league table is depicted in Supplementary Figure S6.

The ranking of the efficacy of the interventions was based on SUCRA, and protocol PRE1 ranked the highest, followed by protocol PREPO5. No protocols were statistically significantly more effective compared to the placebo. A pairwise meta-analysis was conducted for studies with direct comparison and is shown in Supplementary Figure S7. No protocols were statistically significant.

### 3.4. Postsurgical Complications

A total of twelve RCTs investigating 10 interventions have reported postsurgical complications, and a network plot was formed among those studies as shown in Supplementary Figure S8. Based on the NMA, no protocols were statistically significantly effective compared to placebo/no treatment. The SUCRA ranking curve for each protocol is presented in Supplementary Figure S9.

The ranking of the efficacy of the interventions was based on SUCRA, and protocol PREPO7 ranked the highest. The least effective protocol was POST1. No protocols were statistically significantly effective compared to the placebo. Supplementary Table S5 summarizes the RR and the SUCRA ranking of the treatment options.

Based on the SUCRA rank and curve, PREPO7 ranked first while POST1 ranked last in preventing postsurgical complications. Supplementary Figure S10 depicts the league table. For studies with direct comparisons, a pairwise meta-analysis was performed, as shown in Supplementary Figure S11. No protocols were statistically significant.

### 3.5. Adverse Effects

A total of nine RCTs investigating seven interventions reported adverse effects, and a network plot was formed of these studies as shown in Supplementary Figure S12. Based on the NMA, none of the protocols are statistically significant. The direct and network estimates for the side effects of these protocols are shown in Supplementary Table S6.

Based on the SUCRA ranking curve (Supplementary Figure S13), protocol PRE1 ranked first while PREPO2 ranked last in reported adverse effects to antibiotics. Supplementary Figure S14 depicts the league table for adverse effects. A pairwise meta-analysis was conducted for studies with direct comparison and is shown in Supplementary Figure S15. No protocols were statistically significant.

There was no inconsistency found for any outcome of the network meta-analysis (Supplementary Table S7). Based on the comparison-adjusted funnel plot graph, publication bias could be detected for RCTs investigating protocols for implant failure (patient), implant failure (implant), and post-surgical complications (Supplementary Figures S16, S17, and S19). No publication bias was observed for prosthetic failure or adverse effects (Supplementary Figures S18 and S20).

## 4. Discussion

A total of nine out of fifteen randomized controlled trials were deemed to have a high risk of bias using the Cochrane’s Collaboration Tool for assessing risk of bias [41]. Assessors can only predict the susceptibility of a trial to bias, but it will not be known with precision whether there was actual bias during the trial [48]. The inclusion of studies with a high susceptibility to biases without any appropriate handling or additional sensitivity analyses can subject the meta-analysis to being skewed towards favorable or unfavorable outcomes [49]. Thus, this systematic review with network meta-analysis has included a sensitivity analysis with the exclusion of studies with a high risk of bias to justify the results and provide a recommendation for the use of antibiotics in preventing early implant failure.

The results of this network meta-analysis have aligned with previous analyses [16,50,51,52]. Administration of 2 g of amoxicillin 1 h before the surgery had the most significant effect on preventing implant failure across the eleven trials analyzed. Canullo et al. have found that preoperative administration of antibiotics has an associated higher implant survival rate at early stages [51]. This finding is also supported by Roca-Millan et al., Salgado-Peralvo et al., Romandini et al., and Esposito et al. [12,16,50,51]. However, the best antibiotic dosage for short-term prophylaxis remains unknown, and there is insufficient evidence to justify a targeted dosage for short-term prophylaxis. This is shown by the sensitivity analysis, which indicated no significant statistical difference in protocols deployed after the exclusion of studies susceptible to high risk of bias. Furthermore, there was a high degree of heterogeneity in the antibiotic protocols used across the fifteen trials. For example, El-Kholey et al. utilized a 1 g dose of amoxicillin 1 h before surgery protocol, while Nolan et al. used a 3 g dose of amoxicillin instead [27,39]. The majority of studies have included a 2 g dose of amoxicillin 1 h preoperatively with a varied postoperative protocol as shown in Table 1 [28,29,30,32,33,35,36,37,38,40]. Two trials did not report the criteria used for implant failure [32,33]. The definition of implant failure varied from instability when tested with a manual wrench, to mobility of implant, and the presence of any infection. 

Three trials reported the criteria for prosthetic failure, which is when the clinician is unable to place the prostheses on the abutment or secondary to implant failure [35,37,38]. No interventions were significant as compared to no intervention in the prevention of prosthetic failure. Esposito et al. found that the use of antibiotic prophylaxis had only marginal differences compared with no prophylaxis in the prevention of prosthetic failure [52]. 

The results show that none of the antibiotic protocols are statistically significant in the prevention of postsurgical complications. Postsurgical complications have been reported to occur in up to 10% of patients receiving dental implants. Two thirds of the infected implants have to be removed because they do not respond to mechanical or drug therapy [53]. Esposito et al. and Ata-Ali et al. have found that antibiotic prophylaxis does not present statistically significant differences in the prevention of postoperative complications such as infection of the implant [52,54]. Furthermore, this current analysis is also in line with the findings of these previous reviews, which recommend against the use of antibiotics for the purpose of preventing postoperative complications.

This meta-analysis demonstrates that there were no statistically significant differences in adverse effects reported by the nine trials [28,30,31,33,35,36,37,38,40]. Common adverse effects of amoxicillin are gastrointestinal upset, nausea, and vomiting [55]. The greatest concern with the use of antibiotics is anaphylaxis, which can progress to death for the patient, and the increase in antibiotic-resistant strains of bacteria in the oral microbiome of the patient [56,57,58]. In addition to that, resistance to beta-lactams is more prevalent when compared to metronidazole, as demonstrated by Koukos et al. [59]. One study has shown that the use of antibiotic prophylaxis from a patient’s perspective is cost-effective since administration of preoperative antibiotics does have protective effects on preventing early implant failure [60]. However, caution has to be exercised in providing antibiotic prophylaxis from the healthcare perspective, as the emergence of resistant bacteria leads to a significant increase in treatment costs [60]. The development of antibiotic-resistant bacteria along with risks of hypersensitivity outweighs its protective effect against early implant failure. 

The limitations of the current study are the low number of trials with low bias and the high heterogeneity of the antibiotic protocols used. The majority of the interventions varied by duration, dosage, and type of antibiotic. This complicates the analysis, and a standardized selection of protocol should be employed in future trials to determine the best dosage, timing, and type of antibiotic. The authors recommend that future studies be aimed at comparing varied doses of preoperative antibiotic prophylaxis. A different class of antibiotic, such as metronidazole, in the prevention of early implant failure would also be worth investigating due to the anaerobic nature of the microbiome associated with implant failure.

## 5. Conclusions

A single dose of 2 g of preoperative amoxicillin significantly reduces early implant failure in healthy individuals. However, more high-quality trials are required to establish this recommendation as the quality of the evidence is weak. 

## Figures and Tables

**Table 1:** Characteristics of included studies.

Author (Year)	Study Design	Sample Size	Study Comparison	Time of Evaluation	* Outcome: Implant Failure (Patient)	* Outcome: Implant Failure (Implant)	* Outcome: Prosthetic Failure (Patient)	* Outcome: Prosthetic Failure (Implant)	* Outcome: Adverse Effect	* Outcome: Postsurgical Complication
Momand et al. (2022) [28]	Double-blinded, RCT	473	PRE1, PLA	Postsurgical complication and adverse effect: 14 days; implant: 3–6 months	6/238; 7/235	8/373; 8/384	nil	nil	0/238; 0/235	2/238; 5/235
Reza Tabrizi et al. (2022) [32]	Single-blind, single center clinical trial RCT	450	PRE1, PREPO3, and POST3	Three months	0/150; 2/150; 1/150	0/150; 2/150; 1/150	nil	nil	nil	9/150; 11/150; 7/150
Payer et al. (2020) [26]	RCT	236	PREPO1, PLA	Twelve weeks	3/117; 1/119	3/117; 1/119	nil	nil	nil	nil
Kashani et al. (2019) [34]	Randomized clinical trial	447	PRE1, PLA	Four months	11/223; 29/224	12/535; 32/428	nil	nil	nil	nil
Andrade et al. (2017) [33]	RCT	66	PRE1, PREPO2	One, two, three, and seventh day postop, as well as 3 months	0/35; 3/31	nil	nil	nil	0/35; 4/31	nil
Arduino et al. (2015) [35]	Two centered RCT	343	PRE1, PREPO5	Postsurgical complication: 7 days, implant: 1–2 months	5/166; 5/177	5/278; 8/289	0/166; 2/177	0/not stated; 4/not stated	0/166; 3/177	6/166; 4/177
Rory Nolan et al. (2014) [39]	Double-blinded RCT	55	PLA, PRE3	Postsurgical complication: 7 days, implant: 3–4 months	0/27; 5/28	nil	nil	nil	nil	0/27; 2/28
El-Kholey et al. (2014) [27]	Pilot study	80	PRE2, PREPO6	Implant failure and postsurgical complications	0/40; 0/40;	0/47; 0/43	nil	nil	nil	3/40; 1/40
Tan, Wan Ching et al. (2014) [29]	Multicenter RCT	329	PLA, PRE1, PREPO1, and POST1	Eight weeks	0/81; 0/82; 0/86; 1/80	nil	nil	nil	nil	0/81; 1/82; 1/86; 0/80
Ashraf Abukaraky (2011) [36]	Quasi-random controlled clinical trial	240	PRE1, PREPO9, and POST2	One week, one month, and the beginning of the prosthetic stage (after 3–4 months)	12/73; 11/79; 13/88	14/210; 13/266; 15/290	nil	nil	0/73; 2/79; 0/88	1/73; 3/79; 1/88
Caiazzo, Alfonso (2011) [40]	Prospective, con- trolled, and randomized clinical study	100	PLA, PRE1, PREPO8, and POST3	Postsurgical complications and adverse effects: first, second, fourth, and eighth week. Implant: 3 months	nil	0/35; 0/36; 0/48;2/29	nil	nil	0/25; 0/25; 0/25; 0/25	0/25; 0/25; 0/25; 0/25
Esposito, Marco et al. (2010) [38]	Pragmatic multicenter placebo-controlled randomized clinical trial	506	PLA, PRE1	First and second weeks, and the fourth month	5/252; 12/254	7/489; 13/483	4/252; 10/254	nil	0/252; 0;254	8/252; 11/254
Anitua, Eduardo (2009) [30]	Double-blinded RCT	105	PLA, PRE1	Three days, ten days, one month, and three months	2/52; 2/53	2/52; 2/53	nil	nil	0/52; 0/53	6/52; 6/53
Abu-Ta’a et al. (2008) [31]	RCT	80	PLA, PREPO7	Ten days	0/40; 3/40	0/128; 5/119	nil	nil	0/40; 0/40	1/40; 4/40
Esposito, Marco et al. (2008) [37]	Double-blinded RCT	316	PLA, PRE1	One week, two weeks, and four months	2/158; 8/158	nil	2/158;4/158	2/not stated; 4/not stated	1/158; 1/158	7/158; 4/158

Abbreviations: PLA = placebo/no antibiotics; PRE1 = 2 g amox 1 h pre-op; PRE3 = 3 g amox 1 h pre-op; POST1 = 2 g amox immediately post-op; POST2 = post-op amox with clavulanic acid 625 mg 3 times daily for 5 days; POST3 = 500 mg q 8 h for 5 days post-op/postoperative amox 1 g twice a day for 1 week after surgery; PREPO1 = 2 g amox pre-op and 500 mg amox post-op for 3 days; PREPO2 = 2 g amox 1 h pre-op followed by 500 mg three times for 7 days; PREPO3 = 2 g amox 1 h pre-op, 500 mg q 8 h for 5 days; PREPO5 = 2 g amox pre-op and 1 g amox postoperative, and 1 g twice a day for 2 days later; PREPO7 = 1 g amox 1 h pre-op and 500 mg 4x daily for 2 days post-op; PREPO9 = 2 g amox 1 h pre-op, followed by postoperative 500 mg thrice for 5 days. * The numbers x/xxx in the column denote the number of outcomes reported/the sample size in that group.

**Table 2:** Results of network meta-analysis: implant failure (per patient).

Treatment	Implant Failure (Patient)		
	Relative Risk (95% CI)	*p*-Value	SUCRA Rank (Score)
POST1	0.47 (0.02; 10.74)	0.640	7 (55.1)
POST2	0.38 (0.16; 0.90)	0.028 *	4 (64.0)
POST3	1.28 (0.05; 32.30)	0.879	8 (35.6)
PRE1	0.42 (0.27; 0.67)	0 *	6 (59.6)
PRE3	0.11 (0; 1.92)	0.132	1 (82.4)
PREPO1	1.42 (0.22; 8.96)	0.704	9 (26.9)
PREPO2	3.37 (0.17; 65.03)	0.421	11 (18.1)
PREPO3	2.14 (0.10; 45.74)	0.626	10 (24.6)
PREPO5	0.40 (0.10; 1.47)	0.170	5 (60.6)
PREPO7	0.14 (0; 2.67)	0.193	2 (77.1)
PREPO9	0.36 (0.15; 0.87)	0.024 *	3 (66.3)

Note: presence of (*) and texts in red indicate statistically significant protocols. Abbreviations: PRE1 = 2 g amox 1 h pre-op; PRE3 = 3 g amox 1 h pre-op; POST1 = 2 g amox immediately post-op; POST2 = post-op amox with clavulanic acid 625 mg 3 times daily for 5 days; POST3 = 500 mg q8h for 5 days post-op/postoperative amox 1 g twice a day for 1 week after surgery; PREPO1 = 2 g amox pre-op and 500 mg amox post-op for 3 days; PREPO2 = 2 g amox 1 h pre-op followed by 500 mg three times for 7 days; PREPO3 = 2 g amox 1 h pre-op, 500 mg q8h for 5 days; PREPO5 = 2 g amox pre-op and 1 g amox postoperative, and 1 g twice a day for 2 days later; PREPO7 = 1 g amox 1 h pre-op and 500 mg 4x daily for 2 days post-op; PREPO9 = 2 g amox 1 h pre-op, followed by postoperative 500 mg thrice for 5 days.

**Table 3:** Results of network meta-analysis: implant failure (per implant).

Treatment	Implant Failure (Per Implant)		
	Relative Risk (95% CI)	*p*-Value	SUCRA Rank (Score)
POST2	0.38 (0.11,1.31)	0.127	4 (62.6)
POST3	0.43 (0.04,4.16)	0.468	5 (56.3)
PRE1	0.49 (0.26,0.92)	0.027 *	6 (54.2)
PREPO1	3.05 (0.28,33.09)	0.359	9 (11.8)
PREPO3	0.99 (0.07,13.61)	0.998	8 (34.4)
PREPO5	0.76 (0.17,3.39)	0.721	7 (38.1)
PREPO7	0.08 (0,1.68)	0.106	1 (85.0)
PREPO8	0.23 (0.01,4.84)	0.346	2 (68.3)
PREPO9	0.36 (0.10,1.25)	0.110	3 (64.3)

Note: presence of (*) and text in red indicate statistically significant protocols. Abbreviations: PRE1 = 2 g amox 1 h pre-op; POST2 = post-op amox with clavulanic acid, 625 mg 3 times daily for 5 days; POST3 = 500 mg q 8 h for 5 days post-op/postoperative amox, 1 g twice a day for 1 week after surgery; PREPO1 = 2 g amox pre-op and 500 mg amox post-op for 3 days; PREPO3 = 2 g amox 1 h pre-op,500 mg q 8 h for 5 days; PREPO5 = 2 g amox pre-op and 1 g amox postoperative, and 1 g twice a day for 2 days later; PREPO7 = 1 g amox 1 h pre-op and 500 mg 4x daily for 2 days post-op; PREPO8 = 2 g amox 1 h pre-op, followed by postoperative 1 g amox twice a day for 7 days; PREPO9 = 2 g amox 1 h pre-op, followed by postoperative 500 mg thrice for 5 days.

## Data Availability

Data provided in the supplementary material.

## Supplementary Materials

The following supporting information can be downloaded at: https://www.mdpi.com/article/10.3390/antibiotics12030512/s1, Figure S1: Pairwise meta-analysis: Implant failure (per patient data); Figure S2: Sensitivity Analysis Network Plot: Implant failure (per patient data); Figure S3: Sensitivity Analysis Network Plot: Implant failure (per implant data); Figure S4: Network plot: Prosthetic failure (Patient); Figure S5: SUCRA ranking curve for Prosthetic Failure (Patient); Figure S6: League table for Prosthetic Failure (Patient); Figure S7: Pairwise meta-analysis for Prosthetic Failure (Patient); Figure S8: Network plot: Postsurgical Complications; Figure S9: SUCRA ranking curve for Postsurgical Complications; Figure S10: League table for Postsurgical Complication; Figure S11: Pairwise meta-analysis for Postsurgical complications; Figure S12: Network plot: Adverse Effect; Figure S13: SUCRA ranking curve for Adverse Effect; Figure S14: League table for Adverse Effect; Figure S15: Pairwise meta-analysis for Adverse Effects; Figure S16: Comparison-adjusted funnel plot for Implant failure (Patient); Figure S17: Comparison-adjusted funnel plot for Implant failure (per implant data); Figure S18: Comparison-adjusted funnel plot for prosthetic failure; Figure S19: Comparison-adjusted funnel plot for postsurgical complications; Figure S20: Comparison-adjusted funnel plot for adverse effects; Table S1: Abbreviations; Table S2: Risk of bias assessment of included studies; Table S3: Results of network meta-analysis from sensitivity analysis: implant failure (per patient data); Table S4: Results of network meta-analysis: Prosthetic failure (Patient); Table S5: Results of network meta-analysis: Postsurgical Complications; Table S6: Results of network meta-analysis: adverse effect; Table S7: Network consistency for implant failure (patient), implant failure (implant), prosthetic failure (patient), postsurgical complications and adverse effects. References [26,27,28,29,30,31,32,33,34,35,36,37,38,39,40] are cited in Supplementary Materials.
