# Supplementary material for: Comparative Efficacy of Different Amoxicillin Dosing Regimens in Preventing Early Implant Failure—A Systematic Review with Network Meta-Analysis
Source: Antibiotics (Basel). 2023 Mar 3;12(3):512. doi: 10.3390/antibiotics12030512 (PMC10044278; doi:10.3390/antibiotics12030512)

Supplementary Table S1. Abbreviations

| Abbreviation | Full text |
|---|---|
| PLA | placebo/ no antibiotics |
| PRE1 | 2 g amoxicillin 1 hr pre-op |
| PRE2 | 1 g amoxicillin pre-op |
| PRE3 | 3 g amoxicillin 1 hr pre-op |
| POST1 | 2 g amoxicillin immediately post-op |
| POST2 | Post-op amoxicillin with clavulanic acid 625 mg 3x daily for 5 days |
| POST3 | 500 mg q 8 h for 5 days post-op/postoperative amoxicillin, 1 g twice a day for 1 week after surgery |
| PREPO1 | 2 g amoxicillin pre-op and 500 mg amoxicillin post-op for 3 days |
| PREPO2 | 2 g amoxicillin 1 hr pre-op followed by 500 mg three times for 7 days |
| PREPO3 | 2 g amoxicillin 1 hr pre-op, 500 mg q 8 h for 5 days |
| PREPO4 | 2 g amoxicillin 1 hr pre-op followed by placebo post-op |
| PREPO5 | 2 g amoxicillin pre-op and 1 g amoxicillin postoperative, and 1 g twice a day for 2 days later |
| PREPO6 | 1 g amoxicillin pre-op and 500 mg amoxicillin post-op for 3 days |
| PREPO7 | 1 g amoxicillin 1 hr pre-op and 500 mg 4x daily for 2 days post-op |
| PREPO8 | 2 g amoxicillin 1 hr pre-op, followed by postoperative 1 g amoxicillin twice a day for 7 days |
| PREPO9 | 2 g amoxicillin 1 hr pre-op, followed by postoperative 500 mg thrice for 5 days |
| NMA | Network meta-analysis |
| SUCRA | Surface under cumulative ranking |
| RoB | Risk of bias |
| PRISMA | Preferred Reporting Items for Systematic Reviews and Meta-Analyses |
| RR | Relative Risk |
| CI | Confidence Interval |

Supplementary Table S2. Risk of bias assessment of included studies.

| Author | Year | Random Sequence | Allocation Concealment | Blinding of Participants | Blinding of Outcome Assessment (Detection Bias) | Incomplete Outcome | Selective Reporting | Other Bias | Overall Bias |
|---|---|---|---|---|---|---|---|---|---|
| Momand et al. | 2022 | - | - | - | - | - | - | - | - |
| Tabrizi et al. | 2022 | - | - | ? | - | - | - | - | ? |
| Payer et al. | 2020 | - | - | - | - | + | + | - | + |
| Kashani et al. | 2019 | + | ? | + | ? | - | - | - | + |
| Andrade et al. | 2017 | - | ? | ? | ? | - | ? | ? | ? |
| Arduino et al. | 2015 | - | - | + | + | + | - | - | + |
| Nolan et al. | 2014 | - | - | - | - | + | - | - | + |
| Tan et al. | 2014 | - | - | - | - | - | - | - | - |
| El-Kholey et al. | 2014 | - | - | - | - | - | + | - | + |
| Karaky et al. | 2011 | + | + | + | - | + | ? | + | + |
| Caiazzo et al. | 2011 | - | ? | + | + | - | - | + | + |
| Esposito et al. | 2010 | - | - | - | - | - | - | - | - |
| Anitua et al. | 2009 | - | - | - | - | - | - | - | - |
| Abu-Ta'a et al. | 2008 | - | - | + | - | - | - | ? | + |
| Esposito et al. | 2008 | - | - | - | - | - | - | + | + |

Supplementary Table S3. Results of network meta-analysis from sensitivity analysis: implant failure (per patient data)

| Treatment | Implant failure (patient) | | |
|---|---|---|---|
| | Relative risk (95% CI) | P-value | SUCRA rank (score) |
| POST1 | 0.32 (0.01,7.86) | 0.490 | 1 (73.8) |
| POST3 | 1.89 (0.07,49.63) | 0.702 | 4 (41.1) |
| PRE1 | 0.63 (0.31,1.26) | 0.192 | 3 (68.0) |
| PREPO1 | 0.31 (0.12,7.50) | 0.472 | 2 (73.6) |
| PREPO2 | 4.96 (0.24,100.29) | 0.296 | 6 (20.3) |
| PREPO3 | 3.15 (0.14,70.43) | 0.469 | 5 (25.4) |

Abbreviations: PLA = placebo/no antibiotics; PRE1 = 2 g amox 1 hr pre-op; POST1 = 2 g amox immediately post-op; POST3 = 500 mg q 8 h for 5 days post-op/postoperative amox 1 g twice a day for 1 week after surgery; PREPO1 = 2g amox pre-op and 500 mg amox post-op for 3 days; PREPO2 = 2 g amox 1 hr pre-op followed by 500 mg three times for 7 days; PREPO3 = 2 g amox 1 hr pre-op,500 mg q 8 h for 5 days

Supplementary Table S4. Results of network meta-analysis: prosthetic failure (patient)

| Treatment | Prosthetic failure (Patient) | | |
|---|---|---|---|
| | Relative risk (95% CI) | P-value | SUCRA rank (score) |
| PRE1 | 0.43 (0.16,1.11) | 0.082 | 1 (89.7) |
| PREPO5 | 2.02 (0.08,48.38) | 0.663 | 2 (25.4) |

Abbreviations: PRE1 = 2 g amox 1 hr pre-op; PREPO5 = 2 g amox pre-op and 1 g amox postoperative, and 1 g twice a day for 2 days later

Supplementary Table S5. Results of network meta-analysis: postsurgical complications

| Treatment | Postsurgical Complications | | |
|---|---|---|---|
| | Relative risk (95% CI) | P-value | SUCRA rank (score) |
| POST1 | 2.27 (0.21,24.20) | 0.495 | 10 (22.6) |
| POST2 | 0.61 (0.04,8.53) | 0.714 | 4 (59.6) |
| POST3 | 0.75 (0.25,2.20) | 0.604 | 5 (57.0) |
| PRE1 | 0.90 (0.52,1.57) | 0.734 | 6 (49.2) |
| PRE3 | 0.20 (0.01,4.12) | 0.302 | 2 (80.2) |
| PREPO1 | 1.89 (0.24,14.35) | 0.537 | 8 (26.9) |
| PREPO3 | 1.14 (0.41,3.12) | 0.797 | 7 (37.7) |
| PREPO5 | 0.56 (0.14,2.22) | 0.417 | 3 (66.9) |
| PREPO7 | 0.25 (0.02,2.13) | 0.206 | 1 (81.1) |

| | | | |
|---|---|---|---|
| PREPO9 | 1.90 (0.27,13.02) | 0.511 | 9 (25.2) |

Abbreviations: PRE1 = 2 g amox 1 hr pre-op; PRE3 = 3 g amox 1 hr pre-op; POST1 = 2 g amox immediately post-op; POST2 = post-op amox with clavulanic acid 625 mg 3x daily for 5 days; POST3 = 500 mg q 8 h for 5 days post-op/postoperative amox 1 g twice a day for 1 week after surgery; PREPO1 = 2 g amox pre-op and 500 mg amox post-op for 3 days; PREPO3 = 2 g amox 1 hr pre-op, 500 mg q 8 h for 5 days; PREPO5 = 2 g amox pre-op and 1 g amox postoperative, and 1 g twice a day for 2 days later; PREPO7 = 1 g amox 1 hr pre-op and 500 mg 4x daily for 2 days post-op; PREPO9 = 2 g amox 1 hr pre-op, followed by postoperative 500 mg thrice for 5 days

Supplementary Table S6. Results of network meta-analysis: adverse effect

| Treatment | Adverse effect | | |
|---|---|---|---|
| | Relative risk (95% CI) | P-value | SUCRA rank (score) |
| POST2 | -0.18 (-4.46,4.09) | 0.934 | 2 (64.7) |
| PRE1 | 0 (-1.74,1.75) | 0.998 | 1 (66.5) |
| PREPO1 | 0 (-4.25,4.26) | 0.999 | 3 (61.9) |
| PREPO2 | 2.31 (-1.05,5.68) | 0.178 | 7 (21.1) |
| PREPO5 | 1.88 (-1.54,5.31) | 0.282 | 6 (27.4) |
| PREPO7 | 0 (-3.89,3.89) | 1.000 | 4 (59.8) |
| PREPO9 | 1.53 (-1.95,5.02) | 0.389 | 5 (32.3) |

Abbreviations: PRE1 = 2 g amox 1 hr pre-op; POST2 = post-op amox with clavulanic acid 625 mg 3x daily for 5 days; PREPO1 = 2 g amox pre-op and 500 mg amox post-op for 3 days; PREPO2 = 2 g amox 1 hr pre-op followed by 500 mg three times for 7 days; PREPO5 = 2 g amox pre-op and 1 g amox postoperative, and 1 g twice a day for 2 days later; PREPO7 = 1 g amox 1 hr pre-op and 500 mg 4x daily for 2 days post-op; PREPO9 = 2 g amox 1 hr pre-op, followed by postoperative 500 mg thrice for 5 days.

Supplementary Table S7. Network consistency for implant failure (patient), implant failure (implant), prosthetic failure (patient), postsurgical complications, and adverse effects

| Category | Testing for inconsistency | |
|---|---|---|
| | Chi-square ($\chi 2$) | Prob > $\chi 2$ |
| Implant failure (patient) | 4.54 | 0.1033 |
| Implant failure (implant) | 1.53 | 0.4645 |
| Prosthetic failure (patient) | 1.00 | 0.3172 |
| Postsurgical complications | 0.51 | 0.9722 |
| Adverse effects | 1.56 | 0.2120 |

Supplementary Figure S1. Pairwise meta-analysis: implant failure (per patient data)

| Study | exp(ES) | [95% conf. | interval] | % weight |
|---|---|---|---|---|
| Group: POST.. PLA | | | | |
| 6 | 0.325 | 0.013 | 7.869 | 1.14 |
| exp(theta) | 0.325 | 0.013 | 7.869 | |
| Group: PRE1.. P~2 | | | | |
| 7 | 1.113 | 0.541 | 2.288 | 13.38 |
| exp(theta) | 1.113 | 0.541 | 2.288 | |
| Group: PRE1.. P~3 | | | | |
| 2 | 0.333 | 0.014 | 8.118 | 1.13 |
| exp(theta) | 0.333 | 0.014 | 8.118 | |
| Group: PRE1.. PLA | | | | |
| 3 | 0.381 | 0.195 | 0.744 | 14.55 |
| 5 | 0.032 | 0.002 | 0.564 | 1.40 |
| 9 | 0.420 | 0.150 | 1.175 | 8.35 |
| 10 | 1.019 | 0.149 | 6.968 | 2.94 |
| 11 | 0.250 | 0.054 | 1.159 | 4.39 |
| 13 | 0.846 | 0.289 | 2.481 | 7.81 |
| exp(theta) | 0.429 | 0.251 | 0.733 | |
| Group: PRE3.. P~1 | | | | |
| 5 | 2.929 | 0.059 | 144.156 | 0.77 |
| exp(theta) | 2.929 | 0.059 | 144.156 | |
| Group: PRE3.. PLA | | | | |
| 5 | 0.094 | 0.005 | 1.625 | 1.41 |
| exp(theta) | 0.094 | 0.005 | 1.625 | |
| Group: PREP.. P~1 | | | | |
| 6 | 0.954 | 0.019 | 47.527 | 0.76 |
| exp(theta) | 0.954 | 0.019 | 47.527 | |
| Group: PREP.. PLA | | | | |
| 6 | 0.310 | 0.013 | 7.510 | 1.14 |
| 12 | 3.051 | 0.322 | 28.913 | 2.20 |
| exp(theta) | 1.301 | 0.149 | 11.352 | |
| Group: PREP.. P~1 | | | | |
| 15 | 7.875 | 0.423 | 146.697 | 1.34 |
| exp(theta) | 7.875 | 0.423 | 146.697 | |
| Group: PREP.. P~3 | | | | |
| 2 | 1.667 | 0.223 | 12.459 | 2.71 |
| exp(theta) | 1.667 | 0.223 | 12.459 | |
| Group: PREP.. P~1 | | | | |
| 2 | 5.000 | 0.242 | 103.277 | 1.25 |
| exp(theta) | 5.000 | 0.242 | 103.277 | |
| Group: PREP.. P~1 | | | | |
| 4 | 0.938 | 0.277 | 3.181 | 6.41 |
| exp(theta) | 0.938 | 0.277 | 3.181 | |
| Group: PREP.. PLA | | | | |
| 14 | 0.143 | 0.008 | 2.679 | 1.33 |
| exp(theta) | 0.143 | 0.008 | 2.679 | |
| Group: PREP.. P~2 | | | | |
| 7 | 0.943 | 0.448 | 1.982 | 12.90 |
| exp(theta) | 0.943 | 0.448 | 1.982 | |
| Group: PREP.. P~1 | | | | |
| 7 | 0.847 | 0.399 | 1.800 | 12.69 |
| exp(theta) | 0.847 | 0.399 | 1.800 | |
| Overall | | | | |
| exp(theta) | 0.700 | 0.495 | 0.990 | |

Supplementary Figure S2. Sensitivity analysis network plot : implant failure (per patient data)

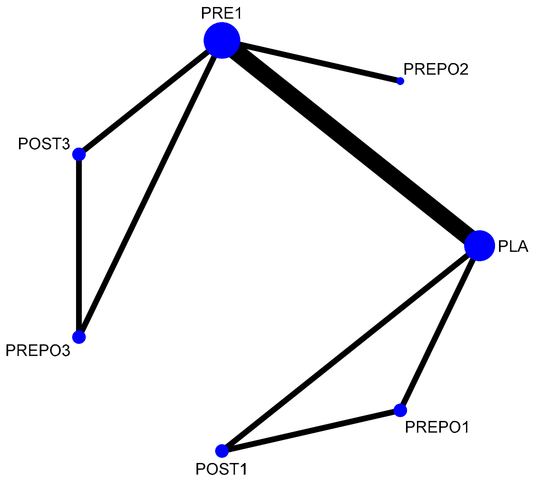


Abbreviations: PLA = placebo/no antibiotics; PRE1 = 2 g amox 1 hr pre-op; POST1 = 2 g amox immediately post-op; POST3 = 500 mg q 8 h for 5 days post-op/postoperative amox 1 g twice a day for 1 week after surgery; PREPO1 = 2 g amox pre-op and 500 mg amox post-op for 3 days; PREPO2 = 2 g amox 1 hr pre-op followed by 500 mg three times for 7 days; PREPO3 = 2 g amox 1 hr pre-op, 500 mg q 8 h for 5 days

Supplementary Figure S3. Sensitivity analysis network plot : implant failure (per implant data)

| Study | exp(ES) | [95% conf. | interval] | % weight |
|---|---|---|---|---|
| Group: POST.. PLA | | | | |
| 8 | 0.122 | 0.006 | 2.465 | 1.31 |
| exp(theta) | 0.122 | 0.006 | 2.465 | |
| Group: PRE1.. P~2 | | | | |
| 7 | 1.289 | 0.636 | 2.612 | 13.66 |
| exp(theta) | 1.289 | 0.636 | 2.612 | |
| Group: PRE1.. P~3 | | | | |
| 2 | 0.333 | 0.014 | 8.118 | 1.17 |
| 8 | 1.361 | 0.028 | 66.990 | 0.79 |
| exp(theta) | 0.587 | 0.050 | 6.931 | |
| Group: PRE1.. PLA | | | | |
| 3 | 0.300 | 0.156 | 0.575 | 14.90 |
| 8 | 0.167 | 0.008 | 3.339 | 1.32 |
| 9 | 0.532 | 0.214 | 1.322 | 10.01 |
| 10 | 1.019 | 0.149 | 6.968 | 3.01 |
| 13 | 1.029 | 0.390 | 2.715 | 9.18 |
| exp(theta) | 0.506 | 0.284 | 0.901 | |
| Group: PREP.. PLA | | | | |
| 12 | 3.051 | 0.322 | 28.913 | 2.26 |
| exp(theta) | 3.051 | 0.322 | 28.913 | |
| Group: PREP.. P~3 | | | | |
| 2 | 1.667 | 0.223 | 12.459 | 2.77 |
| exp(theta) | 1.667 | 0.223 | 12.459 | |
| Group: PREP.. P~1 | | | | |
| 2 | 5.000 | 0.242 | 103.277 | 1.29 |
| exp(theta) | 5.000 | 0.242 | 103.277 | |
| Group: PREP.. P~1 | | | | |
| 4 | 1.539 | 0.510 | 4.648 | 7.59 |
| exp(theta) | 1.539 | 0.510 | 4.648 | |
| Group: PREP.. PLA | | | | |
| 14 | 0.085 | 0.005 | 1.513 | 1.42 |
| exp(theta) | 0.085 | 0.005 | 1.513 | |
| Group: PREP.. P~3 | | | | |
| 8 | 1.324 | 0.027 | 65.204 | 0.79 |
| exp(theta) | 1.324 | 0.027 | 65.204 | |
| Group: PREP.. P~1 | | | | |
| 8 | 0.973 | 0.020 | 47.731 | 0.79 |
| exp(theta) | 0.973 | 0.020 | 47.731 | |
| Group: PREP.. PLA | | | | |
| 8 | 0.162 | 0.008 | 3.250 | 1.32 |
| exp(theta) | 0.162 | 0.008 | 3.250 | |
| Group: PREP.. P~2 | | | | |
| 7 | 0.945 | 0.458 | 1.949 | 13.30 |
| exp(theta) | 0.945 | 0.458 | 1.949 | |
| Group: PREP.. P~1 | | | | |
| 7 | 0.733 | 0.352 | 1.526 | 13.11 |
| exp(theta) | 0.733 | 0.352 | 1.526 | |
| Overall | | | | |
| exp(theta) | 0.757 | 0.533 | 1.076 | |

Supplementary Figure S4. Network plot: prosthetic failure (patient)

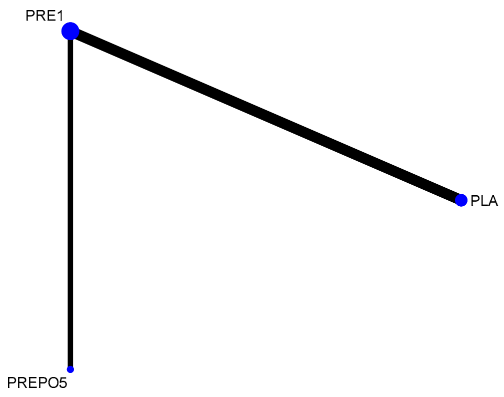


Supplementary Figure S5. SUCRA ranking curve for prosthetic failure (patient)

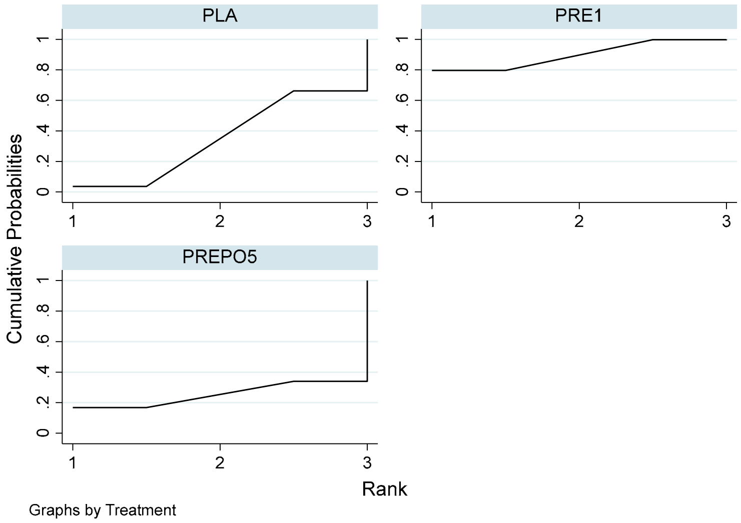


Abbreviations: PLA = placebo/no antibiotics; PRE1 = 2 g amox 1 hr pre-op; PREPO5 = 2 g amox pre-op and 1 g amox postoperative, and 1 g twice a day for 2 days later.

Supplementary Figure S6. League table for prosthetic failure (patient)

| | | |
|---|---|---|
| PRE1 | | |
| 0.21 (0.01,4.41) | PREPO5 | |
| 0.43 (0.17,1.11) | 2.02 (0.08,48.38) | PLA |

Abbreviations: PLA = placebo/no antibiotics; PRE1 = 2 g amox 1 hr pre-op; PREPO5 = 2 g amox pre-op and 1 g amox postoperative, and 1 g twice a day for 2 days later

Supplementary Figure S7. Pairwise meta-analysis for prosthetic failure (patient)

| Study | exp(ES) | [95% conf. | interval] | % weight |
|---|---|---|---|---|
| Group: POST.. PLA | | | | |
| 8 | 0.122 | 0.006 | 2.465 | 1.31 |
| exp(theta) | 0.122 | 0.006 | 2.465 | |
| Group: PRE1.. P~2 | | | | |
| 7 | 1.289 | 0.636 | 2.612 | 13.66 |
| exp(theta) | 1.289 | 0.636 | 2.612 | |
| Group: PRE1.. P~3 | | | | |
| 2 | 0.333 | 0.014 | 8.118 | 1.17 |
| 8 | 1.361 | 0.028 | 66.990 | 0.79 |
| exp(theta) | 0.587 | 0.050 | 6.931 | |
| Group: PRE1.. PLA | | | | |
| 3 | 0.300 | 0.156 | 0.575 | 14.90 |
| 8 | 0.167 | 0.008 | 3.339 | 1.32 |
| 9 | 0.532 | 0.214 | 1.322 | 10.01 |
| 10 | 1.019 | 0.149 | 6.968 | 3.01 |
| 13 | 1.029 | 0.390 | 2.715 | 9.18 |
| exp(theta) | 0.506 | 0.284 | 0.901 | |
| Group: PREP.. PLA | | | | |
| 12 | 3.051 | 0.322 | 28.913 | 2.26 |
| exp(theta) | 3.051 | 0.322 | 28.913 | |
| Group: PREP.. P~3 | | | | |
| 2 | 1.667 | 0.223 | 12.459 | 2.77 |
| exp(theta) | 1.667 | 0.223 | 12.459 | |
| Group: PREP.. P~1 | | | | |
| 2 | 5.000 | 0.242 | 103.277 | 1.29 |
| exp(theta) | 5.000 | 0.242 | 103.277 | |
| Group: PREP.. P~1 | | | | |
| 4 | 1.539 | 0.510 | 4.648 | 7.59 |
| exp(theta) | 1.539 | 0.510 | 4.648 | |
| Group: PREP.. PLA | | | | |
| 14 | 0.085 | 0.005 | 1.513 | 1.42 |
| exp(theta) | 0.085 | 0.005 | 1.513 | |
| Group: PREP.. P~3 | | | | |
| 8 | 1.324 | 0.027 | 65.204 | 0.79 |
| exp(theta) | 1.324 | 0.027 | 65.204 | |
| Group: PREP.. P~1 | | | | |
| 8 | 0.973 | 0.020 | 47.731 | 0.79 |
| exp(theta) | 0.973 | 0.020 | 47.731 | |
| Group: PREP.. PLA | | | | |
| 8 | 0.162 | 0.008 | 3.250 | 1.32 |
| exp(theta) | 0.162 | 0.008 | 3.250 | |
| Group: PREP.. P~2 | | | | |
| 7 | 0.945 | 0.458 | 1.949 | 13.30 |
| exp(theta) | 0.945 | 0.458 | 1.949 | |
| Group: PREP.. P~1 | | | | |
| 7 | 0.733 | 0.352 | 1.526 | 13.11 |
| exp(theta) | 0.733 | 0.352 | 1.526 | |
| Overall | | | | |
| exp(theta) | 0.757 | 0.533 | 1.076 | |

Supplementary Figure S8. Network plot: postsurgical complications

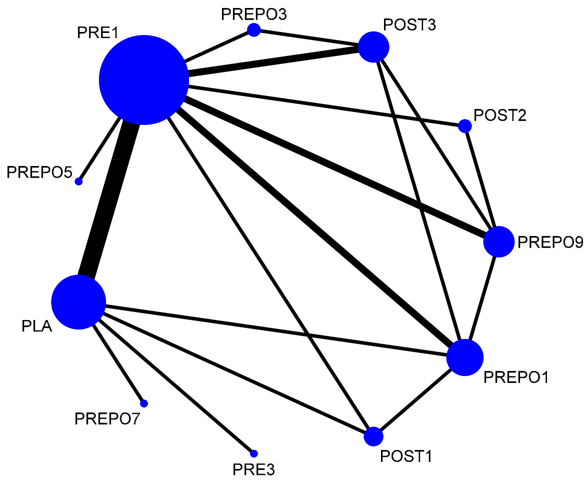


Abbreviations: PLA = placebo/no antibiotics; PRE1 = 2 g amox 1 hr pre-op; PRE3 = 3 g amox 1 hr pre-op; POST1 = 2 g amox immediately post-op; POST2 = post-op amox with clavulanic acid 625 mg 3x daily for 5 days; POST3 = 500 mg q 8 h for 5 days post-op/postoperative amox 1 g twice a day for 1 week after surgery; PREPO1 = 2 g amox pre-op and 500 mg amox post-op for 3 days; PREPO3 = 2 g amox 1 hr pre-op, 500 m g q 8 h for 5 days; PREPO5 = 2 g amox pre-op and 1 g amox postoperative, and 1 g twice a day for 2 days later; PREPO7 = 1 g amox 1 hr pre-op and 500 mg 4x daily for 2 days post-op; PREPO9 = 2 g amox 1 hr pre-op, followed by postoperative 500 mg thrice for 5 days

Supplementary Figure S9. SUCRA ranking curve for postsurgical complications

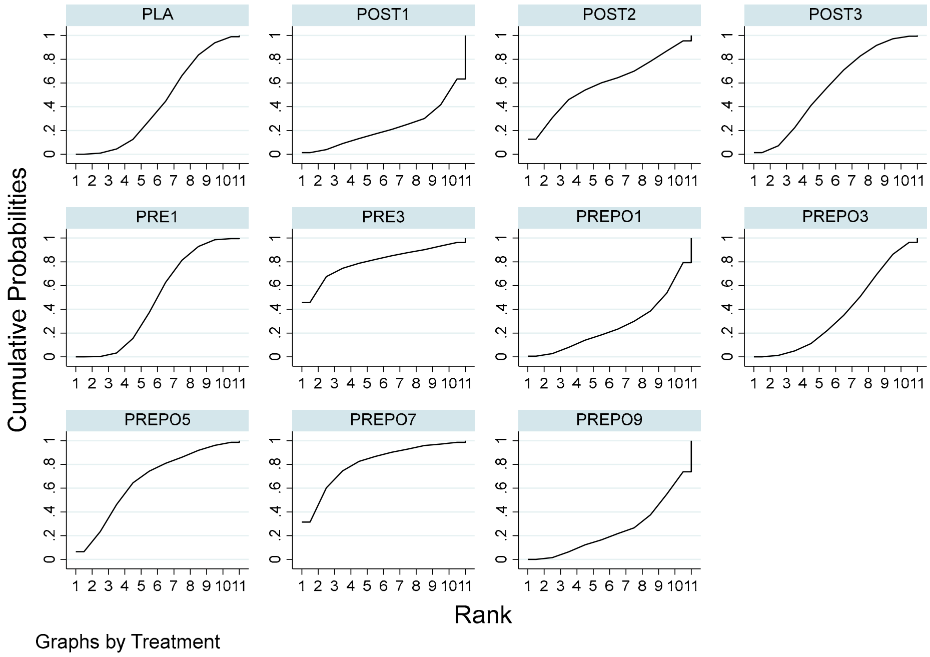


Abbreviations: PLA = placebo/no antibiotics; PRE1 = 2 g amox 1 hr pre-op; PRE3 = 3 g amox 1 hr pre-op; POST1 = 2 g amox immediately post-op; POST2=post-op amox with clavulanic acid 625 mg 3x daily for 5 days; POST3 = 500 mg q 8 h for 5 days post-op/postoperative amox 1 g twice a day for 1 week after surgery; PREPO1 = 2 g amox pre-op and 500 mg amox post-op for 3 days; PREPO3 = 2 g amox 1 hr pre-op, 500 mg q 8 h for 5 days; PREPO5 = 2 g amox pre-op and 1 g amox postoperative, and 1 g twice a day for 2 days later; PREPO7 = 1 g amox 1 hr pre-op and 500 mg 4x daily for 2 days post-op; PREPO9 = 2 g amox 1 hr pre-op, followed by postoperative 500 mg thrice for 5 days

Supplementary Figure S10. League table for postsurgical complications

| | | | | | | | | | | |
|---|---|---|---|---|---|---|---|---|---|---|
| POST1 | | | | | | | | | | |
| 3.73 (0.12,115.22) | POST2 | | | | | | | | | |
| 3.03 (0.25,36.64) | 0.81 (0.05,12.23) | POST3 | | | | | | | | |
| 2.51 (0.24,26.25) | 0.67 (0.05,8.89) | 0.83 (0.33,2.09) | PRE1 | | | | | | | |
| 11.00 (0.24,497.79) | 2.95 (0.05,159.12) | 3.63 (0.15,87.22) | 4.39 (0.21,91.92) | PRE3 | | | | | | |
| 1.20 (0.14,10.41) | 0.32 (0.01,7.58) | 0.40 (0.05,3.39) | 0.48 (0.07,3.54) | 0.11 (0.00,4.06) | PREPO1 | | | | | |
| 2.00 (0.17,23.89) | 0.54 (0.04,7.99) | 0.66 (0.27,1.63) | 0.80 (0.34,1.85) | 0.18 (0.01,4.26) | 1.66 (0.19,14.14) | PREPO3 | | | | |
| 4.01 (0.28,57.28) | 1.07 (0.06,18.92) | 1.32 (0.28,6.25) | 1.60 (0.46,5.57) | 0.36 (0.01,9.77) | 3.33 (0.32,35.11) | 2.01 (0.45,9.05) | PREPO5 | | | |
| 9.11 (0.37,221.98) | 2.44 (0.08,73.28) | 3.01 (0.27,33.19) | 3.64 (0.40,33.37) | 0.83 (0.02,32.93) | 7.57 (0.40,144.99) | 4.57 (0.43,48.91) | 2.27 (0.18,28.93) | PREPO7 | | |
| 1.20 (0.07,21.58) | 0.32 (0.03,2.95) | 0.39 (0.05,2.95) | 0.48 (0.08,3.02) | 0.11 (0.00,3.81) | 0.99 (0.08,12.69) | 0.60 (0.08,4.46) | 0.30 (0.03,2.77) | 0.13 (0.01,2.34) | PREPO9 | |
| 2.28 (0.21,24.21) | 0.61 (0.04,8.54) | 0.75 (0.26,2.20) | 0.91 (0.52,1.58) | 0.21 (0.01,4.13) | 1.89 (0.25,14.36) | 1.14 (0.42,3.12) | 0.57 (0.15,2.22) | 0.25 (0.03,2.14) | 1.91 (0.28,13.02) | PLA |

Abbreviations: PLA = placebo/no antibiotics; PRE1 = 2 g amox 1 hr pre-op; PRE3 = 3 g amox 1 hr pre-op; POST1 = 2 g amox immediately post-op; POST2 = post-op amox with clavulanic acid 625 mg 3x daily for 5 days; POST3 = 500 mg q 8 h for 5 days post-op/postoperative amox 1 g twice a day for 1 week after surgery; PREPO1 = 2 g amox pre-op and 500 mg amox post-op for 3 days; PREPO3 = 2 g amox 1 hr pre-op, 500 mg q 8 h for 5 days; PREPO5 = 2 g amox pre-op and 1 g amox postoperative, and 1 g twice a day for 2 days later; PREPO7 = 1 g amox 1 hr pre-op and 500 mg 4x daily for 2 days post-op; PREPO9 = 2 g amox 1 hr pre-op, followed by postoperative 500 mg thrice for 5 days

Supplementary Figure S11. Pairwise meta-analysis for postsurgical complications

| Study | exp(ES) | [95% conf. | interval] | % weight |
|---|---|---|---|---|
| Group: POST.. PLA | | | | |
| 6 | 2.928 | 0.121 | 70.821 | 1.05 |
| exp(theta) | 2.928 | 0.121 | 70.821 | |
| Group: PRE1.. P~1 | | | | |
| 6 | 0.337 | 0.014 | 8.162 | 1.05 |
| exp(theta) | 0.337 | 0.014 | 8.162 | |
| Group: PRE1.. P~2 | | | | |
| 7 | 1.205 | 0.077 | 18.940 | 1.40 |
| exp(theta) | 1.205 | 0.077 | 18.940 | |
| Group: PRE1.. P~3 | | | | |
| 2 | 1.286 | 0.492 | 3.363 | 11.52 |
| 8 | 1.000 | 0.021 | 48.524 | 0.71 |
| exp(theta) | 1.267 | 0.498 | 3.222 | |
| Group: PRE1.. PLA | | | | |
| 6 | 0.988 | 0.020 | 49.186 | 0.70 |
| 9 | 0.733 | 0.300 | 1.792 | 13.32 |
| 10 | 1.019 | 0.351 | 2.956 | 9.39 |
| 11 | 1.750 | 0.523 | 5.860 | 7.29 |
| 13 | 0.395 | 0.077 | 2.016 | 4.01 |
| exp(theta) | 0.901 | 0.518 | 1.568 | |
| Group: PRE3.. PLA | | | | |
| 5 | 0.207 | 0.010 | 4.126 | 1.19 |
| exp(theta) | 0.207 | 0.010 | 4.126 | |
| Group: PREP.. P~1 | | | | |
| 6 | 0.954 | 0.101 | 8.989 | 2.12 |
| exp(theta) | 0.954 | 0.101 | 8.989 | |
| Group: PREP.. P~3 | | | | |
| 8 | 1.000 | 0.021 | 48.524 | 0.71 |
| exp(theta) | 1.000 | 0.021 | 48.524 | |
| Group: PREP.. P~1 | | | | |
| 6 | 2.828 | 0.117 | 68.428 | 1.05 |
| 8 | 1.000 | 0.021 | 48.524 | 0.71 |
| exp(theta) | 1.861 | 0.159 | 21.845 | |
| Group: PREP.. PLA | | | | |
| 6 | 2.793 | 0.115 | 67.587 | 1.05 |
| exp(theta) | 2.793 | 0.115 | 67.587 | |
| Group: PREP.. P~3 | | | | |
| 2 | 1.571 | 0.626 | 3.944 | 12.57 |
| exp(theta) | 1.571 | 0.626 | 3.944 | |
| Group: PREP.. P~1 | | | | |
| 2 | 1.222 | 0.522 | 2.863 | 14.69 |
| exp(theta) | 1.222 | 0.522 | 2.863 | |
| Group: PREP.. P~1 | | | | |
| 4 | 0.625 | 0.180 | 2.176 | 6.84 |
| exp(theta) | 0.625 | 0.180 | 2.176 | |
| Group: PREP.. PLA | | | | |
| 14 | 0.250 | 0.029 | 2.140 | 2.31 |
| exp(theta) | 0.250 | 0.029 | 2.140 | |
| Group: PREP.. P~2 | | | | |
| 7 | 3.342 | 0.355 | 31.475 | 2.12 |
| exp(theta) | 3.342 | 0.355 | 31.475 | |
| Group: PREP.. P~3 | | | | |
| 8 | 1.000 | 0.021 | 48.524 | 0.71 |
| exp(theta) | 1.000 | 0.021 | 48.524 | |
| Group: PREP.. P~1 | | | | |
| 7 | 2.772 | 0.295 | 26.058 | 2.12 |
| 8 | 1.000 | 0.021 | 48.524 | 0.71 |
| exp(theta) | 2.149 | 0.309 | 14.961 | |
| Group: PREP.. PR~1 | | | | |
| 8 | 1.000 | 0.021 | 48.524 | 0.71 |
| exp(theta) | 1.000 | 0.021 | 48.524 | |
| Overall | | | | |
| exp(theta) | 1.069 | 0.771 | 1.481 | |

Supplementary Figure S12. Network plot: adverse effect

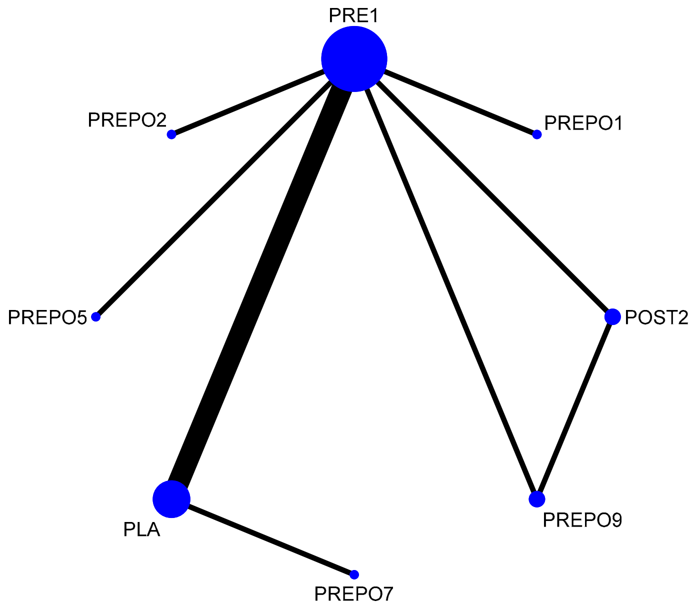


Abbreviations: PLA = placebo/no antibiotics; PRE1 = 2 g amox 1 hr pre-op; POST2 = post-op amox with clavulanic acid 625 mg 3x daily for 5 days; PREPO1 = 2 g amox pre-op and 500 mg amox post-op for 3 days; PREPO2 = 2 g amox 1 hr pre-op followed by 500 mg three times for 7 days; PREPO5 = 2 g amox pre-op and 1 g amox postoperative, and 1 g twice a day for 2 days later; PREPO7 = 1 g amox 1 hr pre-op and 500 mg 4x daily for 2 days post-op; PREPO9 = 2 g amox 1 hr pre-op, followed by postoperative 500 mg thrice for 5 days

Supplementary Figure S13. SUCRA ranking curve for adverse effect

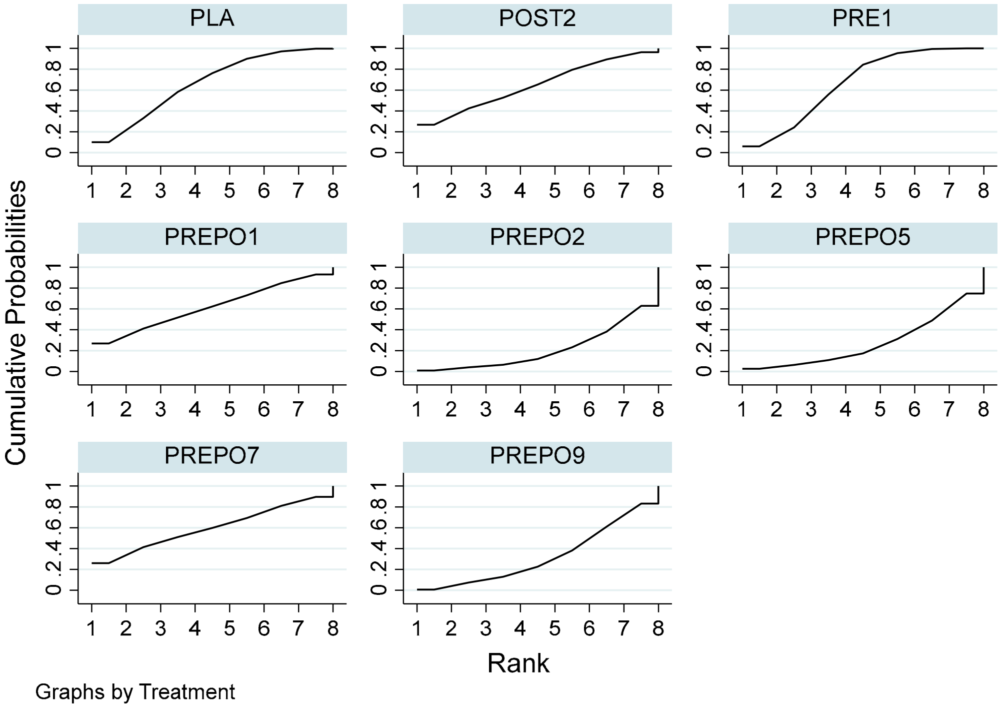


Abbreviations: PLA = placebo/no antibiotics; PRE1 = 2 g amox 1 hr pre-op; POST2 = post-op amox with clavulanic acid 625 mg 3x daily for 5 days; PREPO1 = 2 g amox pre-op and 500 mg amox post-op for 3 days; PREPO2 = 2 g amox 1 hr pre-op followed by 500 mg three times for 7 days; PREPO5 = 2 g amox pre-op and 1 g amox postoperative, and 1 g twice a day for 2 days later; PREPO7 = 1 g amox 1 hr pre-op and 500 mg 4x daily for 2 days post-op; PREPO9 = 2 g amox 1 hr pre-op, followed by postoperative 500 mg thrice for 5 days

Supplementary Figure S14. League table for adverse effect

| | | | | | | | |
|---|---|---|---|---|---|---|---|
| POST2 | | | | | | | |
| 0.83 (0.02,41.40) | PRE1 | | | | | | |
| 0.83 (0.00,205.13) | 1.00 (0.02,48.52) | PREPO1 | | | | | |
| 0.08 (0.00,10.55) | 0.10 (0.01,1.76) | 0.10 (0.00,12.43) | PREPO2 | | | | |
| 0.13 (0.00,17.00) | 0.15 (0.01,2.93) | 0.15 (0.00,20.03) | 1.54 (0.02,95.69) | PREPO5 | | | |
| 0.83 (0.00,272.19) | 1.00 (0.01,71.73) | 1.00 (0.00,321.74) | 10.15 (0.06,1753.57) | 6.58 (0.04,1185.73) | PREPO7 | | |
| 0.18 (0.01,3.69) | 0.22 (0.01,4.43) | 0.22 (0.00,29.57) | 2.19 (0.03,142.31) | 1.42 (0.02,97.14) | 0.22 (0.00,40.29) | PREPO9 | |
| 0.83 (0.01,60.29) | 1.00 (0.17,5.76) | 1.00 (0.01,70.83) | 10.15 (0.35,295.47) | 6.58 (0.21,204.13) | 1.00 (0.02,49.20) | 4.64 (0.14,151.95) | PLA |

Abbreviations : PLA = placebo/no antibiotics; PRE1 = 2 g amox 1 hr pre-op; POST2 = post-op amox with clavulanic acid 625 mg 3x daily for 5 days; PREPO1 = 2 g amox pre-op and 500 mg amox post-op for 3 days; PREPO2 = 2 g amox 1 hr pre-op followed by 500 mg three times for 7 days; PREPO5 = 2 g amox pre-op and 1 g amox postoperative, and 1 g twice a day for 2 days later; PREPO7 = 1 g amox 1 hr pre-op and 500 mg 4x daily for 2 days post-op; PREPO9 = 2 g amox 1 hr pre-op, followed by postoperative 500 mg thrice for 5 days

Supplementary Figure S15. Pairwise meta-analysis for adverse effects

| Study | exp(ES) | [95% conf. | interval] | % weight |
|---|---|---|---|---|
| Group: PRE1.. P~2 | | | | |
| 7 | 1.203 | 0.024 | 59.881 | 6.69 |
| exp(theta) | 1.203 | 0.024 | 59.881 | |
| Group: PRE1.. PLA | | | | |
| 9 | 1.008 | 0.020 | 50.600 | 6.66 |
| 10 | 1.019 | 0.021 | 50.413 | 6.71 |
| 11 | 1.000 | 0.063 | 15.848 | 13.38 |
| 13 | 0.987 | 0.020 | 49.559 | 6.66 |
| exp(theta) | 1.003 | 0.175 | 5.762 | |
| Group: PREP.. P~1 | | | | |
| 8 | 1.000 | 0.021 | 48.524 | 6.78 |
| exp(theta) | 1.000 | 0.021 | 48.524 | |
| Group: PREP.. P~1 | | | | |
| 15 | 10.125 | 0.567 | 180.853 | 12.29 |
| exp(theta) | 10.125 | 0.567 | 180.853 | |
| Group: PREP.. P~1 | | | | |
| 4 | 6.567 | 0.342 | 126.188 | 11.69 |
| exp(theta) | 6.567 | 0.342 | 126.188 | |
| Group: PREP.. PLA | | | | |
| 14 | 1.000 | 0.020 | 49.203 | 6.73 |
| exp(theta) | 1.000 | 0.020 | 49.203 | |
| Group: PREP.. P~2 | | | | |
| 7 | 5.562 | 0.271 | 114.132 | 11.19 |
| exp(theta) | 5.562 | 0.271 | 114.132 | |
| Group: PREP.. P~1 | | | | |
| 7 | 4.625 | 0.226 | 94.759 | 11.20 |
| exp(theta) | 4.625 | 0.226 | 94.759 | |
| Overall | | | | |
| exp(theta) | 2.415 | 0.879 | 6.635 | |

Heterogeneity summary

| Group | df | Q | P > Q | tau2 | % I2 | H2 |
|---|---|---|---|---|---|---|
| PRE1 vs. PO~2 | 0 | 0.00 | . | 0.000 | . | . |
| PRE1 vs. PLA | 3 | 0.00 | 1.000 | 0.000 | 0.00 | 1.00 |
| PREPO1 .. P~1 | 0 | 0.00 | . | 0.000 | . | . |
| PREPO2 .. P~1 | 0 | 0.00 | . | 0.000 | . | . |
| PREPO5 .. P~1 | 0 | 0.00 | . | 0.000 | . | . |
| PREPO7 vs. PLA | 0 | 0.00 | . | 0.000 | . | . |
| PREPO9 .. P~2 | 0 | 0.00 | . | 0.000 | . | . |
| PREPO9 .. P~1 | 0 | 0.00 | . | 0.000 | . | . |
| Overall | 10 | 3.35 | 0.972 | 0.000 | 0.00 | 1.00 |

Test of group differences: Q_b = chi2(7) = 3.35 Prob > Q_b = 0.851

**Supplementary Figure S16.** Comparison-adjusted funnel plot for implant failure (patient)

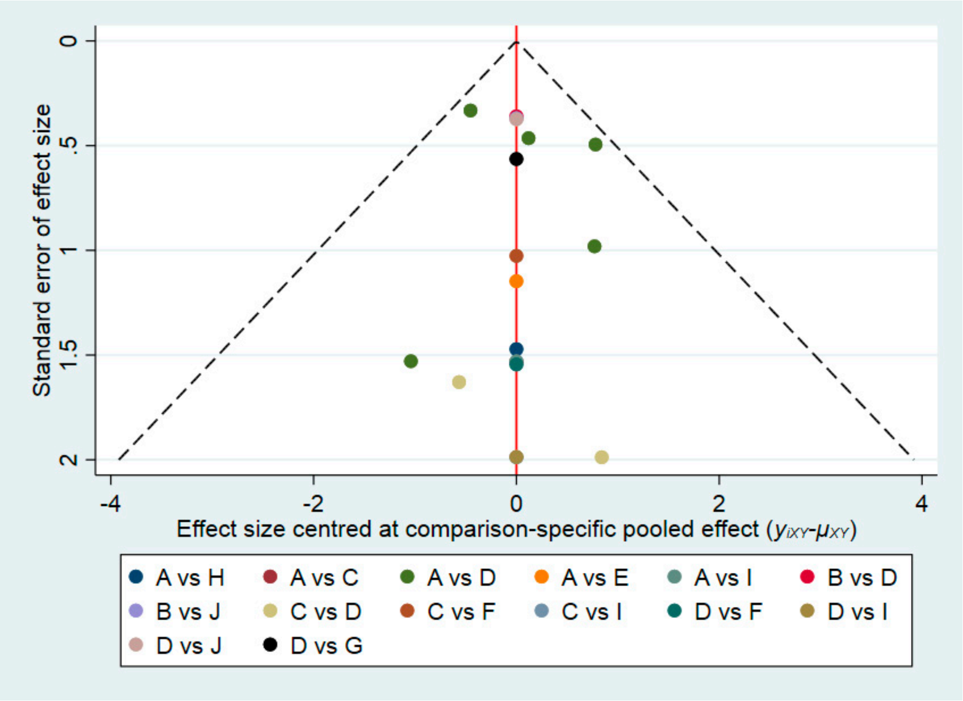


**Supplementary Figure S17**. Comparison-adjusted funnel plot for implant failure (per implant data)

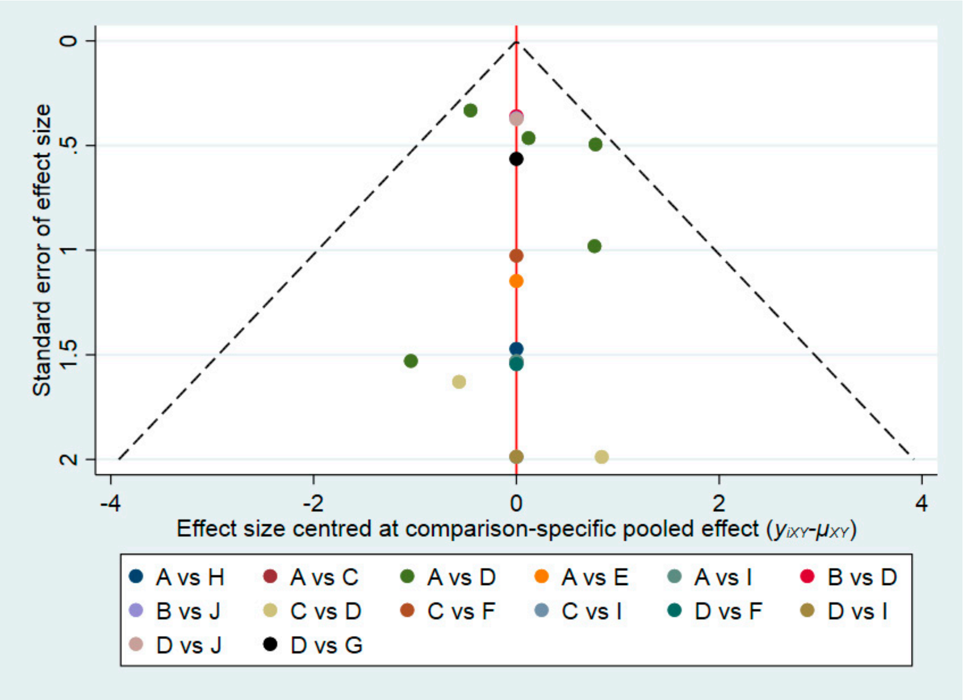

**Supplementary Figure S18**. Comparison-adjusted funnel plot for prosthetic failure

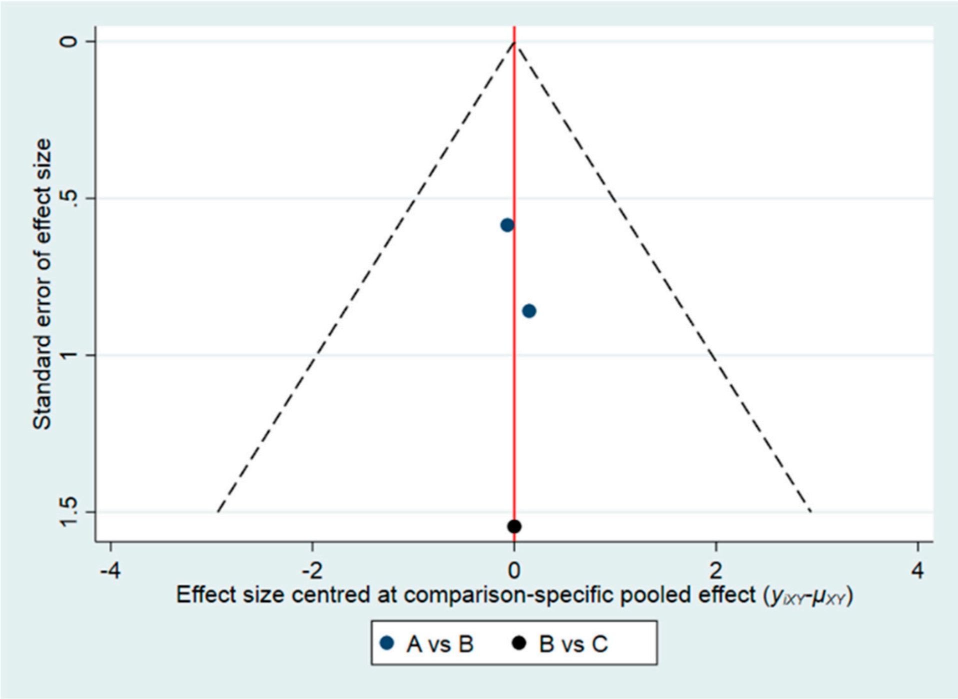


**Supplementary Figure S19**. Comparison-adjusted funnel plot for postsurgical complications

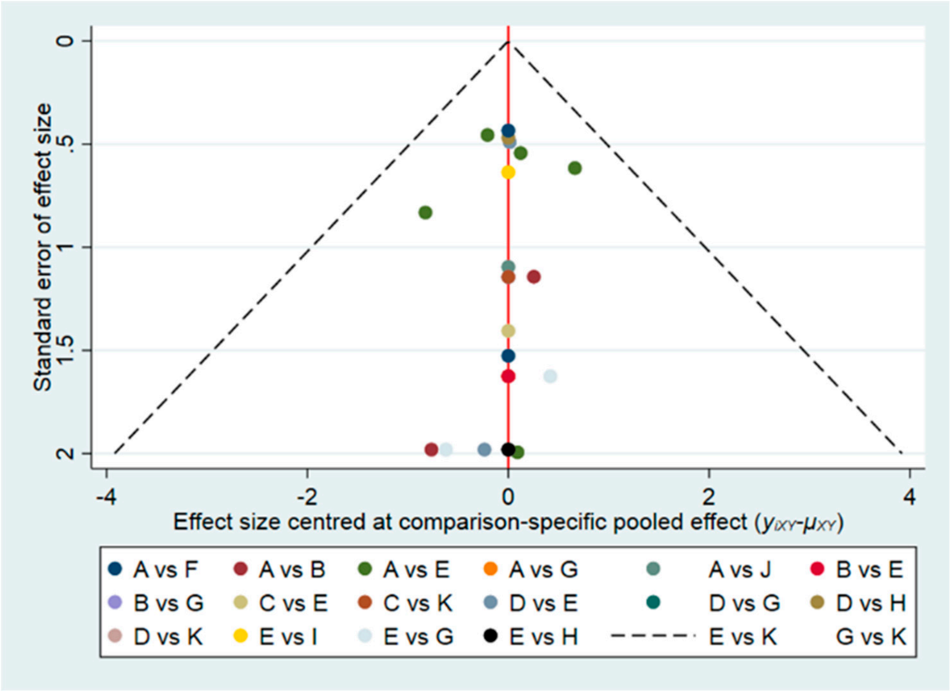

**Supplementary Figure S20**. Comparison-adjusted funnel plot for adverse effects

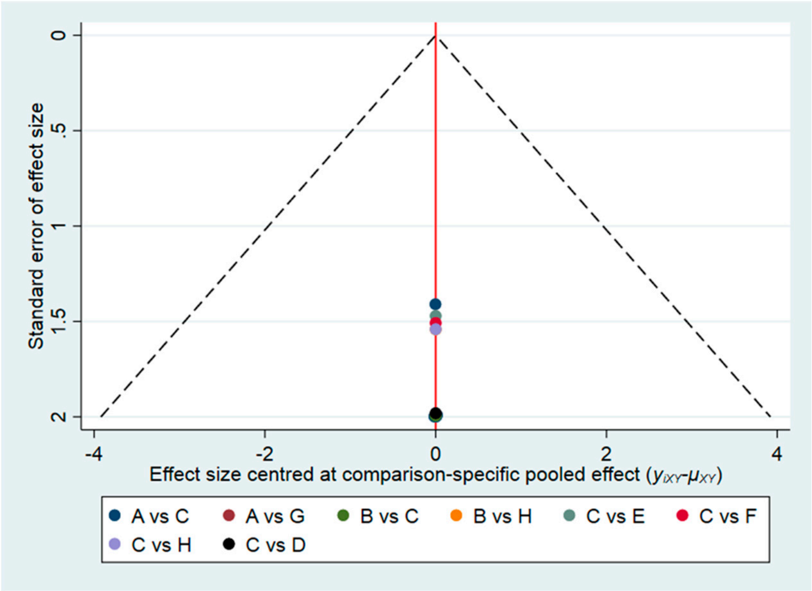

Supplement: Supplementary file 1 [file antibiotics-12-00512-s001.zip › antibiotics-2232733-supplementary.pdf]
